# Knowledge, perception and practices towards sickle cell disease: a community survey among adults in Lubaga division, Kampala Uganda

**DOI:** 10.1186/s12889-018-5496-4

**Published:** 2018-04-27

**Authors:** Sharifu K. Tusuubira, Ritah Nakayinga, Bashir Mwambi, John Odda, Sylvia Kiconco, Alimah Komuhangi

**Affiliations:** 1 Uganda Sickle Cell Rescue Foundation, Plot 4/5 Hotel Close Wampewo Avenue, Clock Tower, P.O. Box 71887, Kampala, Uganda; 2 Clarke International University (formerly International Health Sciences University), St. Barnabas Road, Kisugu- Namuwongo, P.O.Box 7782, Kampala, Uganda

**Keywords:** Sickle cell awareness, Campaigns, KAP, Uganda

## Abstract

**Background:**

Worldwide, the burden of Sickle Cell disease (SCD) has not been amply addressed. In Africa, Uganda has the 5th highest burden, a situation aggravated by limited and inaccessible formal social support structures to aid patients and families cope better with the psychosocial burden of SCD. In addition, this has been coupled with stigmatization and discrimination of people living with sickle cell disease causing isolation from family and society.

**Method:**

This cross sectional study therefore set out to determine the attitudes, perception and level of awareness towards Sickle Cell disease in Ugandan communities. The study used an interviewer administered questionnaires to collect the data.

**Results:**

Out of 110 people sampled; 91.2% of the respondents had ever heard of SCD with the highest proportion 38.7% hearing of SCD from friends and family. Close to half of the respondents 48% knew that SCD is inherited, however a large proportion 44.2% did not know the cause of SCD. However, 68.7% of the respondents said they cannot marry a person with SCD.

**Conclusion:**

The study results indicate that more effort needs to be done to promote sickle cell awareness in Uganda communities with emphasis on the inclusion of sickle cell in health education campaigns.

## Background

Sickle Cell Disease (SCD) is the most common monogenic blood disorder worldwide. It is associated with progressive organ damage coupled with episodes of acute illness [1]. The episodes of acute illness result from the sticky and stiff red blood cells which clog tiny blood vessels. This often results into various conditions not limited to organ and tissue damage, anemia, increased risk of infection and painful episodes [2].

Worldwide, SCD contributes a significant burden that is not amply addressed [3]. It is estimated that 312,000 children will be born worldwide with SCD annually [4]. With the greatest burden existing in Sub-Saharan Africa, where 75% of the world sickle cell disease occurs [5]. In Uganda estimates suggest that 15,000 babies are born each year with sickle cell disease [6]. WHO has indicated the need to improve disease prevention, awareness and early detection in Africa [7, 8].

As a result, the Uganda Sickle Cell Rescue Foundation has been actively championing sickle cell disease awareness and prevention since 2013. Following the Ndeezi et al. 2016 study, the Ministry of Health, Uganda has also made significant strides in addressing the sickle cell disease burden by introducing the newborn screening program in selected districts with the highest disease burden. This increased attention is geared to reducing sickle cell related mortality while increasing care and management outcomes. Sickle cell care and management outcomes are complicated by the complex interaction of SCD patients with the socio-ecological system [9–11]. The dilemma of the person living with sickle cell disease goes beyond grappling with the overwhelming health effects of the disease. The people are often stigmatized and discriminated; this often forces families to hide their sick [12].This survey therefore set out to determine the prevalence, attitudes, perception and knowledge towards Sickle Cell disease in Ugandan adults. This information is crucial in drawing attention to potential areas for intervention and improvement to foster better outcomes in Sickle cell prevention, awareness and management.

## Methods

### Study area

The study was carried out in Lusazze village a peri urban suburb located in Lubaga division, central region of Uganda.

### Study design

The study was a cross-sectional survey. Data were collected between October and September 2016 using a structured questionnaire. The Participants completed a demographic questionnaire which contained information about age, gender, marital status, number of children etc. The other questionnaires were related to knowledge, attitudes and perceptions towards sickle cell disease. Sickle cell screening was also done for participants who provided additional consent. Sickle cell screening was carried out using the solubility test [Biolab, India] [13].

### Sample size and sampling technique

The survey considered a sample size of 110 participants which was obtained using the formula by Kish and Leslie (1965) for cross-sectional studies. [14]. A 95% level of confidence, 50% proportion was estimated and a 5% level of precision were used in the sample size calculation. Convenient sampling was used to select the participants such that all individuals who met the inclusion criteria were included in the survey. Eligible participants were community members who had lived in Lusazze for a period not less than six months. These were interviewed as they came to take part in a sickle cell community outreach.

### Sickle cell community outreach

Uganda Sickle Cell Rescue Foundation carries out awareness activities aimed at having communities of sickle cell disease and its social connotations. Before the outreach announcements are made to inform the community about the event. As part of the event, sickle cell information is shared particularly what sickle cell is, how it is caused and how participants can prevent it. In addition, sickle cell screening is offered so as to enable participants know their genotype.

## Results

### Characteristics of respondents

From Table 1 below, close to two thirds (61.8%) of the respondents are female and in the age group between 19 and 28 years. Slightly more than half (52.9%) of the respondents were not in union (single). The highest proportion of respondents (62.7%) had biological children. More than half (56.9%) of the respondents knew their SCD status.

**Table 1:** Characteristics of respondents

Variable	Frequency (*N* = 102)	Percentage (%)
Sex		
Male	39	38.2
Female	63	61.8
Age group (years)		
< 18	13	12.7
19–28	63	61.8
29–38	20	19.6
> 38	6	5.9
Marital status		
In union	48	47.1
Not in union	54	52.9
Have biological children		
Yes	64	62.7
No	38	37.3
Know your SCD status		
Yes	58	56.9
No	44	43.1

### Knowledge of respondents on sickle cell disease

From Table 2 below, the vast majority (91.2%) of the respondents had ever heard of SCD with the highest proportion (38.7%) of the respondents hearing of SCD from friends and family. Close to half of the respondents (48%) knew that SCD is inherited, however a large proportion (44.2%) did not know the cause of SCD. More than half of the respondents knew some signs and symptoms of SCD. Unfortunately, half of the respondents (50%) did not know how SCD is diagnosed. The highest proportion (45.1%) of the respondents did not know the chance of having a healthy baby when all the parents have SCD. Majority (76.5%) of the respondents noted that conventional medicine was the ideal treatment for SCD.

**Table 2:** Knowledge on Sickle cell disease

Variable	Frequency (*N* = 102)	Percentage (%)
Heard of sickle cell disease		
Yes	93	91.2
No	09	8.8
Source of information (*N* = 93)		
Health professional/ community meetings	11	11.3
TV/ radio	24	25.8
Internet	22	24.2
Friends /family	36	38.7
Causes of SCD		
Acquired	8	7.8
Inherited	49	48.0
Don’t know	45	44.2
Signs & symptoms		
Frequent illness	53	52.0
Yellow eyes	7	6.9
Don’t know	42	41.1
How SCD is diagnosed		
Blood test	45	44.1
Urine test	6	5.9
Don’t know	51	50.0
SCD prevention		
Genetic counseling	22	21.6
Don’t know	39	38.2
Testing before marriage	41	40.2
Chance of getting a healthy baby when all the parents have SCD		
None of the children	5	4.9
All the children	37	36.3
Half chance of the children	11	10.8
Quarter chance baby will be normal	3	2.9
Don’t know	46	45.1
Medication for people with SCD		
Herbal medicine	7	6.8
Conventional medicine	78	76.5
Prayers	6	5.9
Don’t know	11	10.8

### Respondents’ practice and perception on SCD

From Table 3 below, the vast majority (83.4%) had never tested for SCD, most (90. 2%) of the respondents did not know their partner’s genotype. Most of the respondents (74.3%) reported that they wanted to know their SCD status as their reason to test and the highest proportion (60.8%) noted that knowing their SCD status influenced or can influence their decision to marry while more than two thirds (68.7%,) of the respondents cannot marry a person with SCD. When asked whether people with SCD can work, more than two thirds 70% of the respondents reported that they can work while out of those who reported that they cannot work; half reported that being very weak is the reason as to why they shouldn’t work while the other 50% reported that they are often ill so they do not have to work.

**Table 3:** Practice and Perception of SCD

Variable	Frequency (*N* = 102)	Percentage (%)
Have you ever tested for SCD		
Yes	20	19.6
No	82	83.4
Do you know your partner’s genotype		
Yes	10	9.8
No	92	90.2
Reason to test (*n* = 74)		
Curiosity	19	25.7
To know SCD status	55	74.3
Did/ Would knowing your status influence your decision to marry		
Yes	62	60.8
No	40	39.2
Would you marry someone with SCD		
Yes	32	31.7
No	70	68.3
Can someone with SCD work		
Yes	70	68.3
No	32	31.7
If No, why shouldn’t they work (*n* = 32)		
They are very weak	16	50.0
They fall ill very often	16	50.0

## Discussion

This survey presents one of the first SCD community based findings in Lusaze, a semi-urban area in Lubaga division, central region of Uganda. Most SCD campaigns in Uganda generally focus on creating awareness without collecting baseline data.

This survey targeted female and male adults. This campaign attracted a lower proportion of the males. This may be due the fact that most men were away for work or it may also be due to men’s negative attitude as highlighted by Francis et al. 2008 [15] . The study results are similar to Musoke et al. 2014 who also highlighted that more women utilize health facilities/ services in Wakiso District [16]. This may continue to underscore community campaigns since men may be the decision makers in most Ugandan homes.

Most of the participants have heard of SCD which may imply that they know of its existence. However, the fact that only a small proportion obtained such information from a health professional or community meeting may suggest that there is limited effort in health care settings/system to inform the public of SCD. Besides having heard of the disease, a relatively lower proportion had knowledge on the causes, signs and symptoms and prevention and this indicates a much larger problem which may hinder control strategies. Orish et al. 2014 found out that in Ghana schools ranked highest as sources of sickle cell disease knowledge while families ranked highest in our study [17]. Families are often associated with stigma and discrimination coupled with myths / beliefs about sickle cell disease. One participant said ‘*my parents always cautioned us from marrying from a certain family because they always heard somebody hospitalized*’. Families have sometimes been identified as precipitators of stigma and discrimination [18]. The results indicate a percentage of people who consider prayer and herbal medicine as a form of therapy for sickle cell disease yet this was a peri urban locality. This is in contrast to another study where rural Ugandans considered prayer as a form of treatment for chronic diseases [19]. This strengthens the need to promote more health education of sickle cell disease and other chronic illness.

Of note is that a substantial proportion of the participants indicated that they knew their status. When we asked them whether they had tested a greatest proportion had not and indeed the largest proportion had never been screened for SCD. This is because most of them associated sickle cell status with frequent illness which they were free from. This indicates a hefty gap in screening services, yet it may clearly influence family decisions and subsequent control of the disease in the population. Since majority of the participants did not know their partners’ genotype, it may imply that SCD screening before or after marriage is not prioritized yet it may also influence personal or family decisions.

Regarding perception, SCD individuals may be stigmatized and discriminated against since most participants seem to perceive the disease negatively regarding marriage and work. This is similar to what other researchers have found out that individuals with SCD often report SCD related stigma [20, 21].

The study limitations include the small sample size from a single area interviewed over a limited period of time. The small sample size affects our ability to generalize the findings. We recommend future research to interview a larger sample size to be able to validate the findings of this study. The study results could also be affected by the recruitment criteria where participants were selected at the sickle cell community campaign. This could be due to their interest or lack of knowledge about sickle cell disease. The sampling approach utilized is prone to bias, given that this was a community event and the participants were largely self-selected.

## Conclusion

The results show that the respondents have heard of sickle cell mostly from friends and family. Close to half of the respondents knew that SCD is inherited, however a large proportion did not know the cause of SCD. However, more than two thirds of the respondents said they cannot marry a person with SCD.

### Recommendations

There is need for the formulation of strategies to encourage male involvement in SCD campaigns. It is essential for the inclusion of SCD in existing health education programs both at the community and health center settings/levels. The study results indicate that more effort needs to be done to promote sickle cell awareness in Uganda communities.

## Abbreviation

SCD: Sickle Cell Disease
